# Supplementary figures and images for: miRNA Changes in Retinal Ganglion Cells after Optic Nerve Crush and Glaucomatous Damage
Source: Cells. 2021 Jun 22;10(7):1564. doi: 10.3390/cells10071564 (PMC8305746; doi:10.3390/cells10071564)

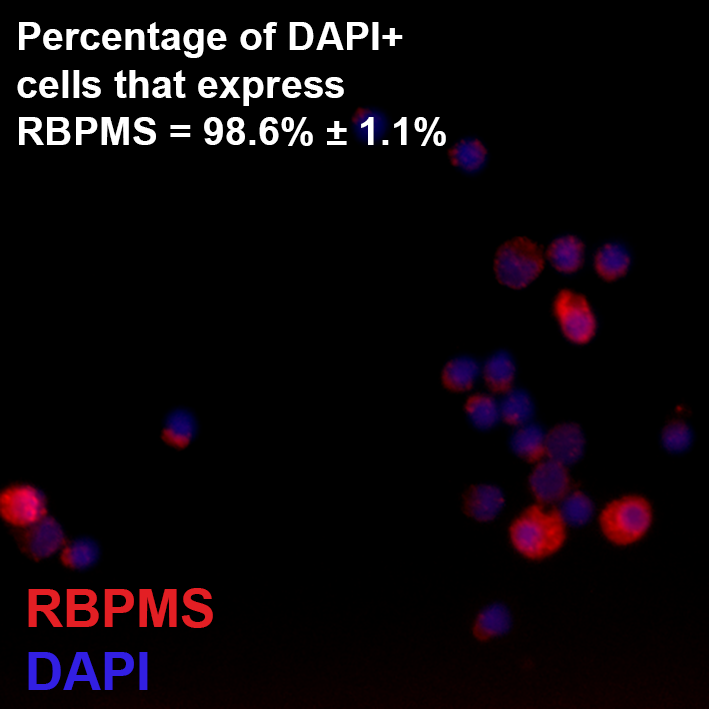

Supplement: Supplementary file 1 [file cells-10-01564-s001.zip › Supplementary Figure S1.tif]
